# Supplementary material for: Longitudinal Nutritional Intakes in Italian Pregnant Women in Comparison with National Nutritional Guidelines
Source: Nutrients. 2022 May 5;14(9):1944. doi: 10.3390/nu14091944 (PMC9101748; doi:10.3390/nu14091944)
Supplement: Supplementary file 1 [file nutrients-14-01944-s001.zip › nutrients-1682738-supplementary.pdf]

Table S1. Frequencies of consumption and ratio factors.

Frequencies of consumption	Ratio Factors
6+ times per day	6.5
4–5 times per day	4.5
2–3 times per day	2.5
1 time per day	1
5–6 times per week	0.786
2–4 times per week	0.429
1 time per month	0.143
1–3 times per month	0.067
Never/<1 time per week	0

Example of calculation of the daily amount of energy, macro and micronutrient: A woman declared to consume whole milk 3 times per week. We multiply the corresponding daily fraction (3/7 = 0.429) by the size portion of the specific food item (expressed as hg—for example, 150 g = 1.5), then by the bromatological composition of food item (energy, macro- and micronutrient items) expressed for 100 grams of product, as reported in the reference database. In this example, the bromatological composition of whole milk provides an energy amount of 64 kcals. Therefore, the final calculation is 0.429 × 1.5 × 64, representing the effective contribution of energy by whole milk to a standard dietary day. The same calculation is repeated for each macro and micronutrient item. In order to obtain the daily amount of energy, macro and micronutrients, we added up the results obtained by this calculation from each food item of the Food Frequency Questionnaire (FFQ).

Table S2. LARN for Energy, SINU 2014.

Height	Weight	BMR	ENERGY REQUIREMENT (kcal/die) FOR A PAL OF:			
**(m)**	**(kg)**	**(kcal/die)**	**1.45**	**1.60**	**1.75**	**2.10**
			Females aged 30–59 years old			
1.50	50.6	1260	1820	2010	2200	2640
1.60	57.6	1310	1900	2100	2300	2760
1.70	65.0	1370	1990	2200	2400	2880
1.80	72.9	1440	2080	2300	2520	3020
1.90	81.2	1510	2180	2410	2630	3160

LARN, Italian pregnant women with Italian nutritional recommendations; SINU, Italian Society of Human Nutrition; BMR, basal metabolic rate; PAL, Physical Activity Level.
